# Effect of Goreisan, a traditional Japanese Kampo medicine, on postoperative nausea and vomiting in gynecological patients

**DOI:** 10.1186/s40981-017-0122-5

**Published:** 2017-09-29

**Authors:** Keiko Kume, Yusuke Kasuya, Makoto Ozaki

**Affiliations:** Department of Anesthesiology, Tokyo Women’s Medical University, 8-1, Kawada-cho, Shinjuku-ku, Tokyo, 162-8666 Japan

**Keywords:** Postoperative nausea and vomiting, Goreisan, Kampo medicine, Gynecological surgery, General anesthesia

## Abstract

**Background:**

Goreisan, a traditional Japanese Kampo medicine, may prevent postoperative nausea and vomiting (PONV). The purpose of this study was to evaluate the effect of Goreisan on PONV in a high-risk population in a randomized, double-blind, placebo-controlled manner.

**Findings:**

Patients undergoing gynecological surgery were randomly allocated to the Goreisan and the control groups. General anesthesia was induced with propofol and remifentanil. After endotracheal intubation, anesthesia was maintained with sevoflurane, fentanyl, and remifentanil. Goreisan 7.5 g dissolved in water (Goreisan group) or water (control group) in a volume of 20 ml was administered through a nasogastric tube approximately 1 h before completion of surgery. The primary outcome of this study was the incidence of PONV during the first 2 h after extubation. In the interim analysis, it was apparent that Goreisan has no effect. Therefore, we discontinued recruiting patients and present results based on data from 83 patients. The incidence of PONV during the first 2 h after extubation was 45% in the Goreisan group (*n* = 40) and 46.5% in the control group (*n* = 43) (*p* = 0.89). There was no significant difference in PONV incidence or severity during the first 24 h post-extubation.

**Conclusion:**

Goreisan has little potency in preventing PONV in high-risk patients.

## Findings

### Introduction

The incidence of postoperative nausea and vomiting (PONV) has been reported to be around 30% [1, 2]. PONV, alongside postoperative pain, is a distress, decreasing patient satisfaction and increasing the risk of dehydration, electrolyte disorder, aspiration pneumonia, prolongation of being bedridden, and delaying discharge [3, 4]. The guidelines for PONV have been updated based on many clinical studies [5, 6]. 5-HT_3_ receptor antagonists, including ondansetron, are the gold standard antiemetics [7, 8], and NK-1 receptor antagonists are also reported to be as effective as 5-HT_3_ receptor antagonists [9, 10]. However, 5-HT_3_ and NK-1 receptor antagonists have not been approved for routine use by the Japanese Ministry of Health, Labor and Welfare as part of the public medical insurance coverage for perioperative antiemetics due to high cost. Only metoclopramide is approved as a perioperative antiemetic in Japan. However, metoclopramide alone is not sufficiently effective to prevent PONV [11]. Because of this Japanese-specific circumstance, a highly effective and low-cost antiemetic with few adverse effects is desired for the prevention of PONV.

Goreisan consists of five herbal galenicals, “Takusha” (*Alismatis rhizoma*), “Bukuryo” (hoelen), “Sojutsu” (*Atractylodis lanceae rhizoma*), “Keihi” (cinnamon bark), and “Chorei” (*Polyporus*), and has been traditionally used as a “hydrostatic modulator” to treat edema, diarrhea, headache, nausea, and dizziness [12]. Although Goreisan increases urine output, like diuretics and furosemide, Goreisan has little effect on the blood electrolyte balance and it does not have diuretic activity in dehydrated individuals. The hydrostatic modulation of Goreisan is considered milder and causes fewer side effects than other diuretics [13]. Goreisan has also been reported to regulate the function of the intestines and show an antiemetic effect [14–16].

In this study, we evaluated the efficacy of Goreisan to prevent PONV in a high-risk patient population undergoing gynecological surgery, with the use of intraoperative volatile anesthetics and postoperative opioids.

### Methods

This prospective, randomized, placebo-controlled clinical study was conducted with the approval of the institutional research board of Tokyo Women’s Medical University and was preregistered as UMIN-000011801 in the University Hospital Medical Information Network Clinical Trials Registry. All patients involved in the study provided written informed consent. Inclusion criteria were patients who (1) underwent elective gynecological surgery, (2) were aged 20 to 50 years, and (3) had an expected procedure time of > 2 h. Exclusion criteria were (1) ASA-PS 3 or more, (2) morbid obesity (body mass index ≥ 35 kg/m^2^), (3) pregnant or lactating women, (4) regular use of any Kampo medicines, and (5) use of steroids, immunosuppressive, or chemotherapy agents.

#### Anesthesia and postoperative protocol

Patients were not given any premedication. General anesthesia was induced with 1–2 mg/kg of propofol, 0.5 μg/kg/min of remifentanil, and 0.6 mg/kg of rocuronium and maintained with sevoflurane to keep the bispectral index (BIS) value between 40 and 60. The remifentanil dose was fixed at 0.5 μg/kg/min throughout the procedure, and total intraoperative fentanyl dose was intended to be 8–12 μg/kg. Postoperative analgesia was established by using an intravenous patient-controlled analgesia pump with fentanyl, at a base dose of 20 μg/h and bolus dose of 20 μg with a 10-min lockout interval.

#### Intervention

After the induction of general anesthesia, patients were assigned to the Goreisan group or control group according to a computer-generated randomization. Assignment was blinded except for the independent investigators who prepared and administered the test drugs.

After the trachea was intubated, a nasogastric tube was inserted and the placement was confirmed by aspiration of gastric fluid. Approximately 1 h prior to the completion of the surgery, the patients in the Goreisan group were administered 7.5 g of Goreisan (Extracts of *Alismatis rhizoma* 4.0 g, Bukuryo 3.0 g*, Atractylodis lanceae rhizoma* 3.0 g, cinnamon bark 1.5 g, and *Polyporus* 3.0 g) dissolved in 20 mL of water at 40 °C. Patients in the control group were administered 20 mL of water at 40 °C as a placebo.

#### Measurements and statistics

PONV just after extubation and at 30 min and 2, 6, and 24 h after extubation were assessed by an anesthesiologist who was blinded the assignment. Nausea severity was rated using a 4-point scale (0: none, 1: slight, 2: moderate, 3: severe). Pain severity was rated using an 11-point scale (0: no pain, 10: severest). Postoperative antiemetic use was also recorded.

The PONV score (smoking history and history of motion sickness or PONV) and fluid disturbance score, which is a criterion for administering Goreisan in traditional Kampo medicine, were assessed preoperatively.

The primary outcome of this study was the incidence of PONV during the first 2 h after extubation. Secondary outcomes were the incidence and severity of PONV at 30 min and 6 and 24 h after extubation and the requirement of antiemetic use.

The unpaired *t* test or Mann-Whitney U-test was used for comparisons of continuous demographic variables, and the chi-square test or Fisher’s exact test was used for nominal variables. A chi-square test and repeated-measures two-way ANOVA were performed to compare the incidence and severity of PONV. The statistical analyses were conducted using JMP® ver.12.1.0 (SAS Institute Inc., Cary, NC). *p* < 0.05 was considered to indicate statistical significance.

#### Sample size estimation

Generally, the incidence of PONV is reported to be 30 to 50%. Since this study population was considered to be at high risk of developing PONV (undergoing gynecological surgery, and use of volatile anesthetics and postoperative opioids), we estimated the baseline PONV incidence seemed to be approximately 50%. As ondansetron, the most effective PONV preventive medicine reduces PONV incidence by 25 to 60% [17–19], we considered that a reduction of 40% in PONV incidence after treatment with Goreisan would be satisfactory. As we calculated that a sample size of 186 patients (93 patients in each) would be needed in order to detect a 40% difference in the incidence of PONV, with an *α* value of 0.05 and a power of 80%, we planned to recruit a total of 200 patients (100 patients in each group), and an interim analysis was planned when the study reached 90 patients.

### Results

**Fig. 1:**
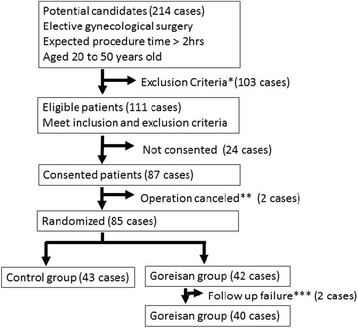
Study flowchart. (One asterisk) Exclusion Criteria: Cardiac disease, liver dysfunction, renal dysfunction, ASA-PS > 3, pregnant, lactating, or current prescription for any Kampo medicine, steroids, immunosuppressive drugs, or anticancer drugs. (Two asterisks) Two cases were removed due to developing a fever or unknown origin on the day of surgery. (Three asterisks) Two cases dropped out due to a technical failure and the inability to obtain follow-up data

After performing the interim analysis, we decided to discontinue the study due to futility. Therefore, this manuscript presents the results of the interim analysis. During the period from February 2014 to March 2015, a total of 470 gynecological surgeries were performed in our institution, and 214 patients among those were considered eligible for the study. After reviewing the medical charts, 103 patients were excluded due to the exclusion criteria, and after an interview, 24 patients did not consent to participate in the study. Two patients were removed from the study after developing a fever of unknown cause after providing informed consent, and in two patients, postoperative data could not be obtained because of a technical follow-up failure. As a result, 83 cases were completed and analyzed (Goreisan group, 40 cases, and control group, 43 cases) (Fig. 1).

The patient demographics did not differ between groups (Table 1). Preoperative PONV risk assessment revealed no significant differences concerning motion sickness, previous PONV history, dizziness, or smoking history (Table 2). The fluid disturbance score was not significantly different between groups (Table 3). Intraoperative and postoperative parameters were shown in Table 4.

**Table 1:** Patient demographics

	Control (*n* = 43)	Goreisan (*n* = 40)	*p* value
Age (years)	38.5 ± 8.0	37.9 ± 8.0	0.73
Body weight (kg)	57.5 ± 10.3	56.9 ± 11.8	0.81
Height (cm)	159.1 ± 5.9	158.9 ± 5.8	0.90
Body mass index (kg/m^2^)	22.5 ± 4.3	22.4 ± 4.9	0.91

Data are expressed as mean ± standard deviation

**Table 2:** Preoperative PONV risk factors

PONV risk factor	Control (*n* = 43)	Goreisan (*n* = 40)	*p* value
Motion sickness	21 (48.8%)	17 (42.5%)	0.57
PONV history	1 (2.3%)	4 (10%)	0.15
Dizziness	9 (20.9%)	12 (30%)	0.35
Smoking history	11(25.6%)	10 (25%)	0.95

Data are expressed as frequencies and percentage

*PONV* postoperative nausea and vomiting

**Table 3:** Fluid disturbance scores

	Score^a^	Control (*n* = 43)	Goreisan (*n* = 40)	*p* value
Heaviness of the body	3	10	12	0.49
Throbbing headache	4	8	7	0.90
Heaviness of the head	3	14	8	0.20
Motion sickness	5	21	18	0.73
Dizziness	5	8	10	0.48
Lightheadedness upon standing	5	10	16	0.1
Watery nasal discharge	3	13	8	0.28
Excessive salivary	3	3	4	0.62
Foamy sputum	4	2	2	0.94
Nausea or vomiting	3	4	1	0.20
Hyperdynamic bowel sounds	3	12	13	0.65
Morning stiffness	7	2	4	0.35
Splashing sound in epigastric region or edematous	15	1	3	0.27
Pleural effusion, cardiac effusion, or ascites	15	0	0	NA
Brisk pulsation in the supra-umbilical region	5	14	15	0.64
Watery diarrhea	5	1	2	0.51
Oliguria	7	0	2	0.14
Polyuria	5	8	14	0.09
Fluid disturbance score		6.5 [4–9.25]	7 [5–11.125]	0.24

Data are expressed as frequencies in each fluid disturbance component, and fluid disturbance score are reported as median with quartile range

^a^The Kampo medicine diagnostic criteria for “fluid disturbance” were evaluated using a simple questionnaire filled by self-assessment. The sum total of each component score is calculated as the fluid disturbance score. If the fluid disturbance score is ≥ 13, the patient is regarded as being in a state of “fluid disturbance.” (If the extent of each symptom is slight, half of the total score was given)

**Table 4:** Intraoperative and postoperative parameters

	Control	Goreisan	*p* value
Pre-operative heart rate (bpm)	75 ± 11	73 ± 10	0.27
Pre-operative sBP (mmHg)	115 ± 16	115 ± 15	0.83
Pre-operative dBP (mmHg)	72 ± 12	71 ± 11	0.88
Pre-operative body temperature (°C)	36.6 ± 0.3	36.7 ± 0.4	0.35
Laparotomy/laparoscopy	21/ 22	24/16	0.31
Procedure time (min)	132 ± 56	131 ± 44	0.93
Anesthesia time (min)	170 ± 60	174 ± 41	0.78
Crystalloid volume (mL)	1439 ± 639	1495 ± 512	0.66
Colloid volume (mL)	23 ± 107	52 ± 220	0.43
Transfusion (mL)	0	28 ± 110	0.1
Urine (mL)	144 ± 186	91 ± 84	0.1
Blood loss (g)	142 ± 274	254 ± 423	0.15
Intra operative fentanyl (μg)	550 ± 98	530 ± 118	0.4
Intra operative remifentanil (mg)	4.1 ± 1.4	4.2 ± 1.2	0.75
Postoperative fentanyl (μg)	802 ± 357	882 ± 382	0.33

Continuous variables reported as mean ± standard deviation, and categorical variable reported as frequencies

*sBP* systolic blood pressure, *dBP* diastolic blood pressure

The cumulative incidence of nausea and vomiting at the time of extubation, at 30 min, at 2 h, and at 24 h after extubation were shown in Table 5. Nausea severity, pain severity, and antiemetic and analgesic use were not different between groups (Tables 6 and 7).

**Table 5:** Accumulative incidence of nausea and vomiting

	Control (*n* = 43)	Goreisan (*n* = 40)	
Incidence of nausea			
At extubation	3 (7.0%)	4 (10%)	0.63
To 30 min	14 (32.6%)	15 (37.5%)	0.64
To 120 min	20 (46.5%)	18 (45%)	0.89
To 6 h	28 (65.1%)	27 (67.5%)	0.82
To 24 h	30 (69.8%)	31 (77.5%)	0.43
Incidence of vomiting			
At extubation	0	0	NA
To 30 min	1 (2.3%)	1 (2.5%)	0.96
To 120 min	5 (11.6%)	3 (7.5%)	0.53
To 6 h	11 (25.6%)	6 (15%)	0.23
To 24 h	21 (48.8%)	23 (57.5%)	0.43

Data are expressed as frequencies and percentage

**Table 6:** Nausea severity and antiemetic use

	Control (*n* = 43)	Goreisan (*n* = 40)	*p* value
Nausea severity			
At extubation	0 [0–0]	0 [0–0]	0.45
To 30 min	0 [0–1]	0 [0–1]	0.84
30 min to 2 h	0 [0–1]	0 [0–0]	0.20
2 h to 6 h	0 [0–2]	0 [0–1.25]	0.82
6 h to 24 h	1 [0–3]	2 [0–3]	0.22
Antiemetic medicine use			
At extubation	0	0	NA
To 30 min	0	1 (2.5%)	0.3
30 min to 2 h	3 (7.0%)	4 (10.0%)	0.62
2 h to 6 h	9 (20.9%)	3 (7.5%)	0.08
6 h to 24 h	18 (41.9%)	15 (41.9%)	0.56

Nausea severity was identified using a 4-point scale (0: none, 1: slightly, 2: moderate, 3: severe). Data in nausea severity are shown as the median and interquartile range

Data in antiemetic medicine use are expressed as frequencies and percentage

**Table 7:** Pain severity and pain relief medication

	Control	Goreisan	*p* value
Pain severity			
At extubation	2 [0–3]	1 [0–4]	0.88
To 30 min	4 [2–7]	5 [3–7]	0.44
30 min to 2 h	3 [2–4]	3 [2–4]	0.90
2 h to 6 h	2 [1–2]	2 [1–3]	0.46
6 h to 24 h	2 [1–3.5]	2 [1–3]	0.26
Pain relief medication			
At extubation	0	0	NA
To 30 min	10 (23.3%)	9 (22.5%)	0.94
30 min to 2 h	5 (11.6%)	4 (10%)	0.81
2 h to 6 h	5 (11.6%)	5 (12.5%)	0.90
6 h to 24 h	12 (27.9%)	11 (27.5%)	0.97

Pain severity was measured using an 11-point scale (0: no pain, 10: severest). Data in pain severity are shown as the median and interquartile range. Data in pain relief medication are expressed as frequencies and percentage

### Discussion

This study was designed to detect the positive difference in PONV incidence between groups; however, study hypothesis was declined. This study showed that Goreisan has no potency in preventing PONV in the first 24 h post-extubation. These results disagree with those of Kori et al. [20]. They administered 7.5 g of Goreisan orally the day before surgery and found that it had a significant effect on PONV. As Goreisan is usually administered 7.5 g daily divided by 3 times (each 2.5 g), authors considered 7.5 g of Goreisan is the almost maximum dose for single administration. We administered 7.5 g of Goreisan dissolved to 20 ml of water intraoperatively through a gastric tube at approximately 1 h before the end of the procedure because Goreisan has a quick onset. However, general anesthesia and laparotomy may reduce digestive tract activity, and the absorption of Goreisan may be delayed. In two of three patients who vomited within 2 h after extubation, the vomit apparently included Goreisan, which has a characteristic color and herbal scent. It indicated that Goreisan may not have been sufficiently absorbed at the end of the procedure in other patients. Even if absorption of Goreisan delayed, because the incidence of PONV in the first 24 h after extubation were similar in the control and Goreisan groups, we can conclude the effect of Goreisan on PONV was clinically negligible.

Although the pharmacological activity of Goreisan has not been fully revealed, we hypothesized that the diuretic effect and drainage of water retained in the interstitial space and improvement of gastrointestinal motility might contribute to preventing PONV [13, 21]. The mechanism of PONV is complex and may include the stimulation of the chemoreceptor trigger zone by residual anesthetic, vestibular nerve disability, diminished peristalsis, elevated digestive tract inner pressure, and psychological reasons. As all of these mechanisms can cause PONV, a single administration of Goreisan is insufficient in preventing PONV in a high-risk population.

We randomized patients without considering the water disturbance score. As the efficacy of Kampo medicines depends on the patient’s individual constitution, diagnostic Kampo medicine-specific examination is very important [12, 22]. In this study, around 30% of patients were identified as being in a state of water disturbance (water disturbance score more than 13). Even if we focused on patients with water disturbance, Goreisan still had no significant effect on PONV.

In this study, Goreisan was given only once during the procedure. In the original traditional Kampo medicine, patients are required to keep taking medicines for about 1 month to ameliorate body constitution. However, practically long time preconditioning before the surgical procedure is not realistic.

This was a negative study on showing PONV prophylactic effect by Goreisan, and showed an incidence of PONV within 24 h in this study population was very high. 5-HT_3_ receptor antagonists, like ondansetron and granisetron which have strong evidence and have been world widely used, should be approved for PONV prophylactic use in Japan. Under limited circumstance like in Japan, conservative methods such as avoiding volatile anesthetics and minimizing postoperative opioid use are the only options to reduce the incidence of PONV in high-risk population. In conclusion, Goreisan has little potency in preventing PONV in high-risk patients.
